# The Hematopoietic Organ: A Cornerstone for *Wolbachia* Propagation Between and Within Hosts

**DOI:** 10.3389/fmicb.2015.01424

**Published:** 2015-12-18

**Authors:** Christine Braquart-Varnier, Maryline Raimond, Gaëtan Mappa, Frédéric D. Chevalier, Winka Le Clec’h, Mathieu Sicard

**Affiliations:** ^1^CNRS UMR 7267, Laboratoire Ecologie et Biologie des Interactions, Université de PoitiersPoitiers, France; ^2^Genetics Department, Texas Biomedical Research Institute, San AntonioTX, USA; ^3^Institut des Sciences de l’Evolution de Montpellier (UMR CNRS-IRD-UM 5554), Université de MontpellierMontpellier, France

**Keywords:** *Wolbachia*, intracellular endosymbiont, hematopoietic organs, hemocytes, terrestrial isopod, symbiosis, mobile vector

## Abstract

*Wolbachia* is an intracellular α-proteobacterium which is transmitted vertically from mother to offspring but also frequently switches horizontally from one host to another. Our hypothesis is based on the role of immune cells and the organs that produce them, the hematopoietic organs (HOs), as primordial niches for the propagation of *Wolbachia via* hemocytes both (i) within hosts: to initiate and maintain the systemic infection and (ii) between hosts: to promote both vertical and horizontal transmission of *Wolbachia*. Therefore, we review some fundamental ideas underlying this hypothesis and go further with new empirical data that lead to a first close-up analysis of the potential role of HOs in *Wolbachia* propagation. The monitoring of the first steps of *Wolbachia* infection in horizontally infected host organs by transmission electron microscopy and qPCR suggests that (i) HOs are colonized early and extensively as soon as they are in contact with *Wolbachia* which find in these cells a favorable niche to multiply and (ii) infected HOs which expel hemocytes all lifelong can generate and maintain a systemic infection that could contribute to increase both vertical and horizontal propagation of these symbionts.

## Introduction

The kind of symbiotic interactions formed between a symbiont and its host depends on the virulence of the first and the resistance of the latter (Combes, 2001). If the symbiont is horizontally transmitted, optimal transmission can be directly linked to virulence as higher multiplication can allow better dispersion. Thus, horizontally transmitted symbionts can maintain high virulence level due to extensive colonization of the host. Contrariwise, vertically transmitted symbionts depend on their host reproduction to transmit. Hence, one could expect an alignment of the interests of both partners that could lead to an attenuated virulence of the symbiont and restriction of symbiont location to gonads. These two extreme situations are observed and documented in nature. However, some symbionts perform both vertical and horizontal transmission in order to optimize their fitness (Ebert, 2013). Such symbiotic systems should present intermediate situation both in terms of colonization and virulence patterns.

*Wolbachia pipientis* is a non-culturable intracellular α-proteobacteria that colonizes ovaries of their arthropod hosts to be vertically transmitted. To increase vertical transmission, *Wolbachia* manipulates its hosts’ reproduction by several ways leading to an increase either in the host female ratio or in fitness for infected females compared to uninfected ones (Werren et al., 2008). In addition to the major vertical transmission path, *Wolbachia* can also quite frequently horizontally pass from one host species to another and adapt to the new host species (Mercot and Poinsot, 2009). Such colonization after horizontal transfer certainly contributes to the large host range of *Wolbachia* which is considered to date as being the most widespread symbiont in the animal kingdom. The most recent meta-analyses of *Wolbachia* pandemics revealed that around 50% of terrestrial arthropod species could be infected with this symbiont (Weinert et al., 2015).

The vertical transmission of *Wolbachia* from mother to offspring is dependent on the symbiont tropism to germ cells (Frydman et al., 2006). However *Wolbachia* frequently exhibits wide intra-host distributions outside of the gonads: they have been observed in the cells of major organs including the brain, nervous chain, muscles, fat body, Malpighian tubules, salivary glands, gnathosoma, midgut and hemolymph in insects, mites and crustaceans (reviewed in Sicard et al., 2014). This intensive extra-gonadal colonization leads to a systemic infection which might be necessary for *Wolbachia* to develop their diverse host manipulations and to spread within host population by other routes than vertical transmission (reviewed in Sicard et al., 2014).

Many terrestrial isopods species were found infected with *Wolbachia* (Bouchon et al., 2008). Phylogenies of both symbionts and hosts appeared incongruent suggesting that *Wolbachia* can transfer horizontally between and within species (Cordaux et al., 2001, 2012; Zimmermann et al., 2015). Such horizontal transfers are readily performed experimentally: (i) by injection of ovary suspension (Rigaud et al., 1991; Juchault et al., 1994; Le Clec’h et al., 2012, 2013, 2014), (ii) by transplantation of infected tissues (Juchault et al., 1974) (iii) *via* hemolymph (injection or hemolymph contact between injured isopods) (Rigaud and Juchault, 1995; Le Clec’h et al., 2014) and (iv) cannibalism (Le Clec’h et al., 2013). Both hemolymphatic contact and cannibalism were closely investigated as potential ecological paths for horizontal transfers between terrestrial isopods (Rigaud and Juchault, 1995; Le Clec’h et al., 2013, 2014). Cannibalism experiments demonstrated that *Wolbachia* was able to pass the intestinal wall and contaminate organs of the predator (Le Clec’h et al., 2013). However, predator exhibited only really low infection loads and the infection was eventually lost for many of them (Le Clec’h et al., 2013). Contrariwise, hemolymph contact led to the systemic infection of all recipient animals which exhibited *Wolbachia* loads similar to vertically infected individuals demonstrating that this path leads to successful horizontal transfers (Rigaud and Juchault, 1995; Le Clec’h et al., 2014).

After introduction of *Wolbachia* in a naïve host by injection of hemolymph or ovary suspension many somatic cells including the hemocytes (i.e., the immune cells) of recipient hosts were extensively infected with *Wolbachia* as observed in naturally infected animals (Braquart-Varnier et al., 2008; Chevalier et al., 2011; Le Clec’h et al., 2014). This observation raises the question of the selective advantage, for *Wolbachia*, of colonizing somatic cells. In *Armadillidium vulgare*, Genty et al. (2014) have hypothesized that many oocytes would be infected secondarily by *Wolbachia* coming from somatic cell sources during isopod life. Indeed, an optimal vertical transmission would not be dependent only on germ line primary tropism but also on the harvesting of some *Wolbachia* cells coming from somatic tissues external to the gonads (Faria and Sucena, 2013; Genty et al., 2014). It is thus possible that *Wolbachia* vertical propagation may rely on distant reservoir requiring mobile vectors, such as hemocytes, to shuttle *Wolbachia* from a source organ to a sink one (i.e., the ovaries) (Rigaud et al., 1991; Rigaud and Juchault, 1995; Chevalier et al., 2011). Hemocytes are good candidates for *Wolbachia* propagation both (i) between organs of an individual and (ii) between individuals during horizontal transfer *via* hemolymph contact. In both terrestrial isopods and insects such as anopheles, *Wolbachia* were spotted in the hemocytes suggesting that the utilization of these cells as “cellular taxis” could be quite common in the arthropod world (Chevalier et al., 2011; Hughes et al., 2011). However, the presence of alive and infectious *Wolbachia* is only demonstrated in the terrestrial isopods. Indeed, the comparison of *Wolbachia* colonization dynamics in terrestrial isopods after the injection of a similar dose of *Wolbachia* from ovary suspension or hemolymph revealed that the latter allowed a quicker colonization of somatic and reproductive organs (Le Clec’h et al., 2014). Such ability of hemocytes to act as “cellular taxis” could be linked to the fact that 40% of them are colonized by around seven *Wolbachia* on average (Chevalier et al., 2011). As the hemocyte density in hemolymph is around 20,000 cells/μL, in the 30 μL of hemolymph contained in an adult, at least 1.10^6^ *Wolbachia* are expected to circulate permanently in the whole host body.

Colonization of immune cells could constitute a primordial step for the propagation of *Wolbachia* both (i) within hosts, to initiate and maintain the systemic infection, and (ii) between hosts, to promote both vertical and horizontal transmission of *Wolbachia*. Addressing the question of the production and renewing of infected immune cells during host life contributes to the understanding of *Wolbachia*-host interactions at both organismal and ecological scales. We present in this paper empirical elements that led to a first close-up analysis of the potential role of Hematopoietic Organs (HOs), involved in the hemocyte production, in *Wolbachia* propagation. To investigate the first step of host colonization after introduction of contaminated hemocytes, we performed injection of hemolymph and HOs transplantations from animals infected with *Wolbachia* to naive ones. The monitoring of the *Wolbachia* infection in host recipient organs by transmission electron microscopy (TEM) and qPCR suggests that (i) HOs are colonized early and extensively as soon as they are in contact with *Wolbachia* which find in these cells a ‘favorable niche’ to multiply and (ii) infected HOs expelling hemocytes all lifelong can then generate and maintain a systemic infection contributing to increase both vertical and horizontal propagation of these symbionts.

## Materials and Methods

### Animals

*Armadillidium vulgare* individuals used in these experiments were either infected with a feminizing *Wolbachia* strain (*w*VulC) (i.e., symbiotic) [lines originating from Helsingör (Denmark) or Celles-sur-Belle (France) (Rigaud et al., 1991; Cordaux et al., 2004)] or uninfected (i.e., asymbiotic) animals [lines originating from Helsingör (Denmark) or Nice (France) (Bouchon et al., 1998; Sicard et al., 2010)]. As *A. vulgare* males are not usually infected with *Wolbachia* (due to the feminizing nature of these *Wolbachia*), only females were used in this study. All animal lines were reared at 20°C under natural photoperiod with food provided *ad libitum*.

### Introduction of *Wolbachia* by Hemolymph Injection

Cuticles of all animals (donors and recipients) used in these experiments were disinfected by immersing individuals for 30 s in a 10% sodium hypochlorite solution followed by a 30 s immersion in distilled water to avoid bacterial contamination from the cuticle. Transinfection was performed by injecting hemolymph, carrying hemocytes infected with *Wolbachia* (∼10^4^ *Wolbachia*/μL). To collect hemolymph, the cuticle of each of symbiotic *A. vulgare* females used as donors was pierced dorsally between the sixth and the seventh pereion segments using a needle and 10 μL of hemolymph from 5 infected females were collected with a micropipette and pooled in a microtube kept on ice, in order to limit coagulation of hemocytes. Then, one microliter of hemolymph was injected into the general cavity of recipient asymbiotic individuals with help of a Hamilton syringe after having pierced laterally the sixth pereion segment.

### Introduction of *Wolbachia* by HOs Transplantation

The HOs which are localized between the sixth and seventh pereion segment and the first telson segment, along the dorsal vessel, were dissected from animals (either infected with *Wolbachia* or not). Then, they were sucked up with a thin glass Pasteur pipette and grafted in the recipient host (either infected with *Wolbachia* or not) for which a small square of 2 mm × 2 mm of cuticle was cut off to allow the introduction of the Pasteur pipette containing the graft.

### *Wolbachia* Quantification in Recipient Hosts

After dissections, total DNA was extracted from the different organs of each recipient individual as described by Kocher et al. (1989). For each sample, the concentration and quality (ratios OD 260/280 nm and 260/230 nm) of the extracted DNA were measured using the Nanodrop 1000 spectrophotometer (Thermo). The quantification of *Wolbachia* was performed by qPCR on DNA samples from ovaries, nervous chain, hemocyte and HO for each treatment collected 15 days after transinfection (either by hemolymph injection or HO transplantation). *Wolbachia* quantification was also performed for all inocula in order to measure the injection dose. All qPCR reactions were performed using a LightCycler 480 system (Roche) as described in Le Clec’h et al. (2012).

### Transmission Electron Microscopy

To analyze the colonization of HOs and other organs by *Wolbachia* using TEM, tissues were fixed (9% glutaraldehyde, 0.3M sodium cacodylate, 3% NaCl, v/v/v) for 2 h at 4°C then washed (0.3M sodium cacodylate, 3% NaCl, 0.8M sucrose, v/v/v) for 2 h at 4°C. Post-fixation was performed in 4% OsO_4_, 0.3M sodium cacodylate, 5.5% NaCl for 45 min at room temperature. Tissues were subsequently dehydrated though a graded series of acetone solutions, infiltrated and embedded in resin (Spurr, Polyscience Inc.). First sections (0.5 μm) were stained with 1% toluidin blue for light microscopy observation. Thinner sections (90 nm) were contrasted by incubation in 1% uranyl acetate in 50% ethanol for 1 min, and then stained with lead citrate. Sections were observed using a transmission electron microscope (JEM 1010).

## Results

### The Trafficking of Hemocytes Into Several Organs

**FIGURE 1:**
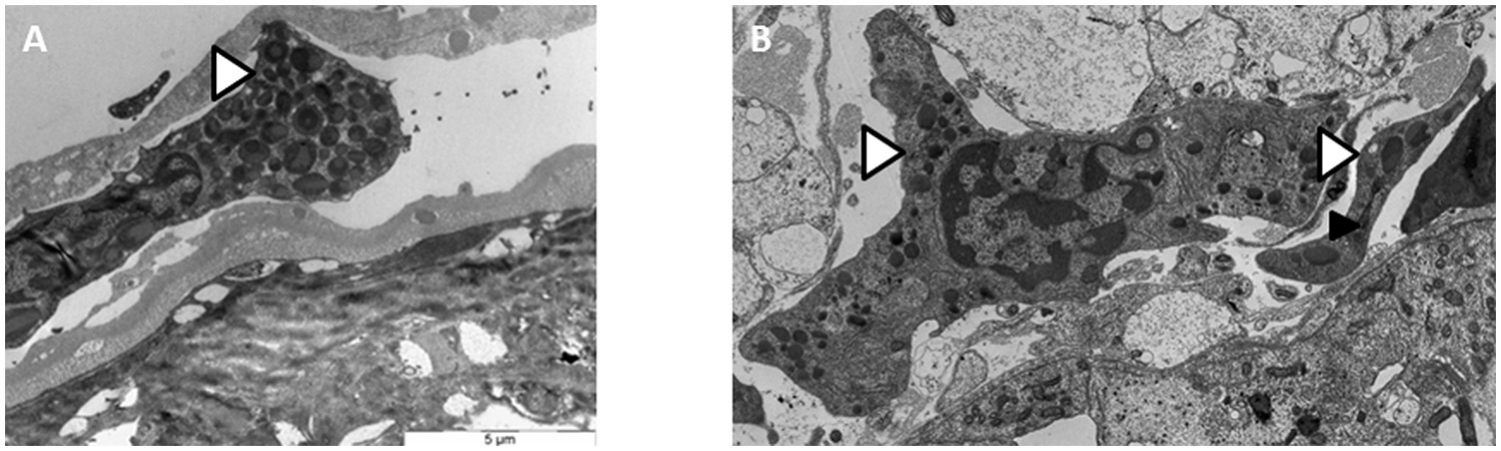
**Transmission electron microscopy (TEM) observations of hemocytes trafficking in the ovary **(A)** and in the nervous chain (**B**).** 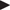 *Wolbachia* 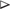 Hemocytes trafficking.

In several *A. vulgare’*s organs, notably in nervous chain and in ovaries, we observed in TEM some hemocytes trafficking directly in the organs. These observations reveal a direct contact between hemocytes and surrounding cells which can promote the passage of *Wolbachia* from one cell type to another (**Figure 1**).

### HOs of Recipient Hosts are Highly Colonized After Horizontal Transfers *Via* Hemolymph Injection or HOs Transplantation

**FIGURE 2:**
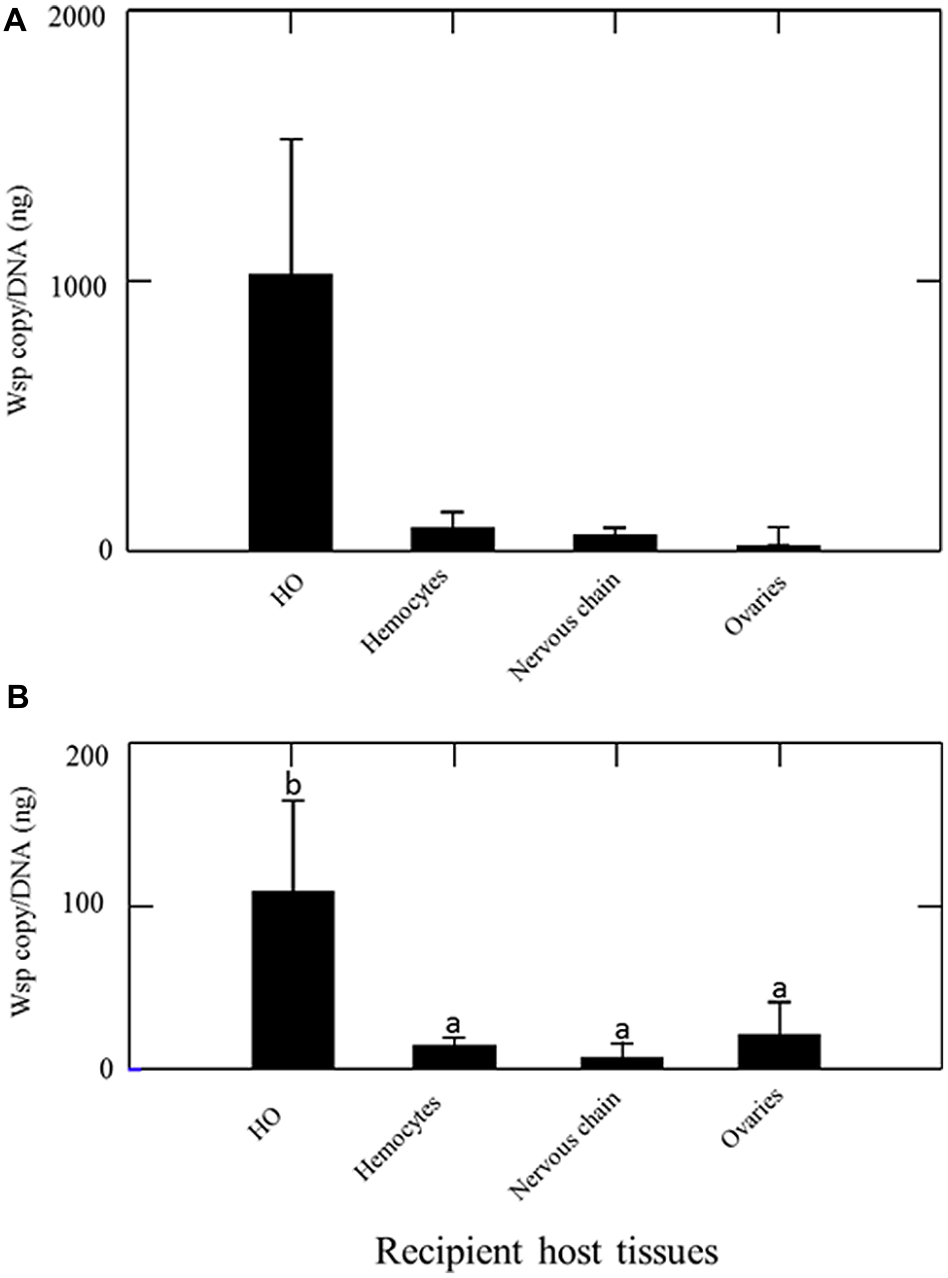
**Quantification of *Wolbachia* [*wsp* copy/DNA (ng)] in asymbiotic recipient animals 15 days after their transinfection **(A)** by injection of hemolymph from symbiotic animals **(B)** by transplantation of HOs from symbiotic animals.** Different letters indicate significant differences.

The quantification of *Wolbachia* by qPCR in 12 asymbiotic recipient animals 15 days after their transinfection with hemolymph from symbiotic animals revealed that all tissues collected (ovaries, nervous chain, hemocytes and HOs) in the recipient animals were infected with *Wolbachia* (**Figure 2A**). The HOs were overall not significantly more colonized than other organs (Kruskal-Wallis test Non-significant). However, the highest densities of *Wolbachia* were registered in HOs (some individuals showed more than 2000 *Wolbachia*/ng DNA in HOs while other tissues never exhibit more than 200 *Wolbachia*/ng DNA in any recipient individuals).

The quantification of *Wolbachia* in nine asymbiotic recipient animals 15 days after they received a HOs transplantation from symbiotic animals revealed that all tissues collected (ovaries, nervous chain, hemocytes, HOs) in the recipient animals were infected with *Wolbachia* (**Figure 2B**). The infection patterns observed is close to the one observed after transinfection with hemolymph (**Figure 2A**). However, in this experiment *Wolbachia* density was significantly higher in the HOs of recipient animals than in any other organ (Kruskal–Wallis: 14,620; *P* = 0.002).

### HOs: A Privileged Environment for *Wolbachia* Multiplication?

**FIGURE 3:**
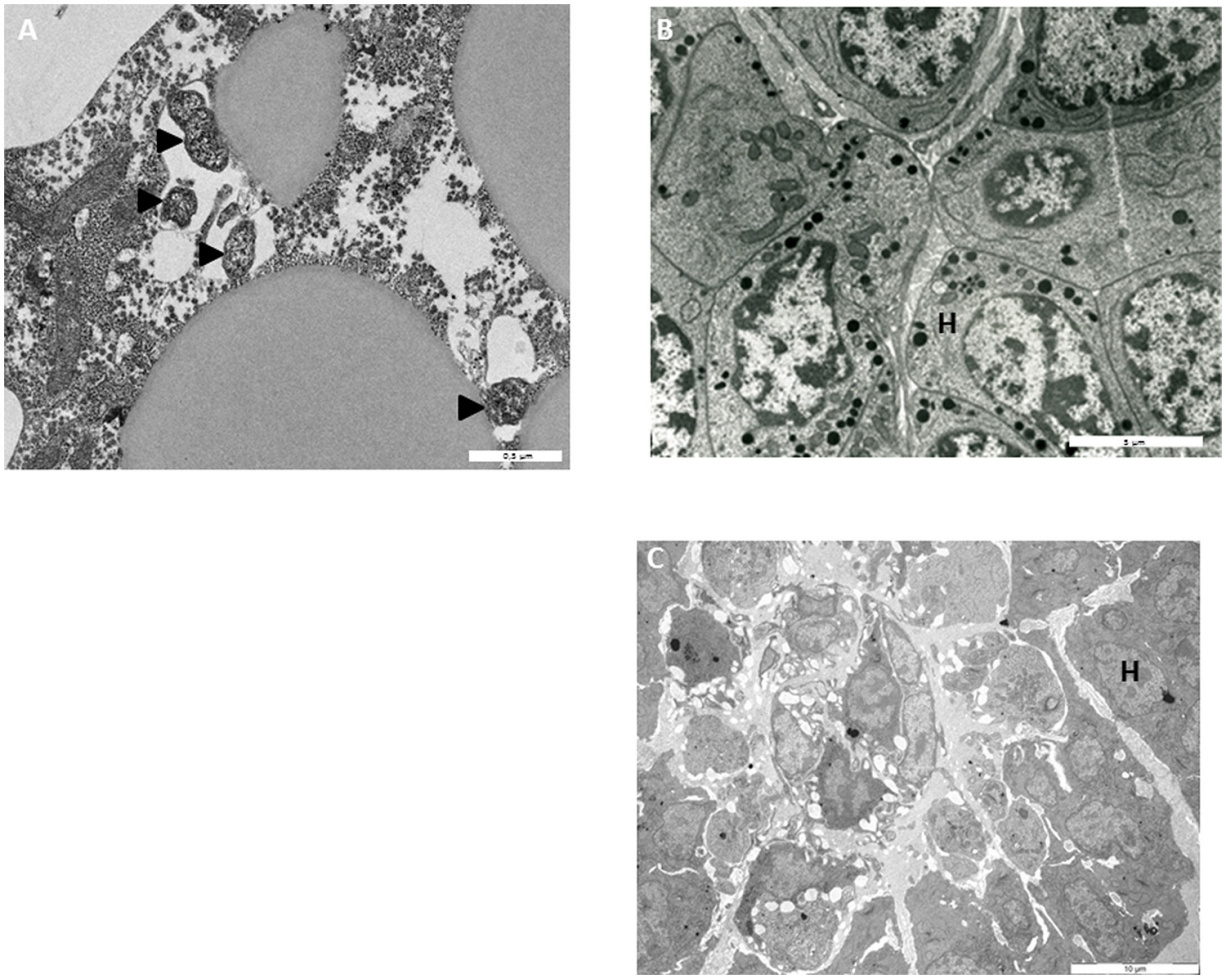
**Transmission electron microscopy observations **(A)***Wolbachia* in adipocyte surrounding grafted HOs 15 days after transplantation; **(B)** HO from asymbiotic animals **(C)** asymbiotic HO grafted in symbiotic environment (15 days after transplantation).** H, hemocytes 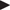 *Wolbachia.*

**FIGURE 4:**
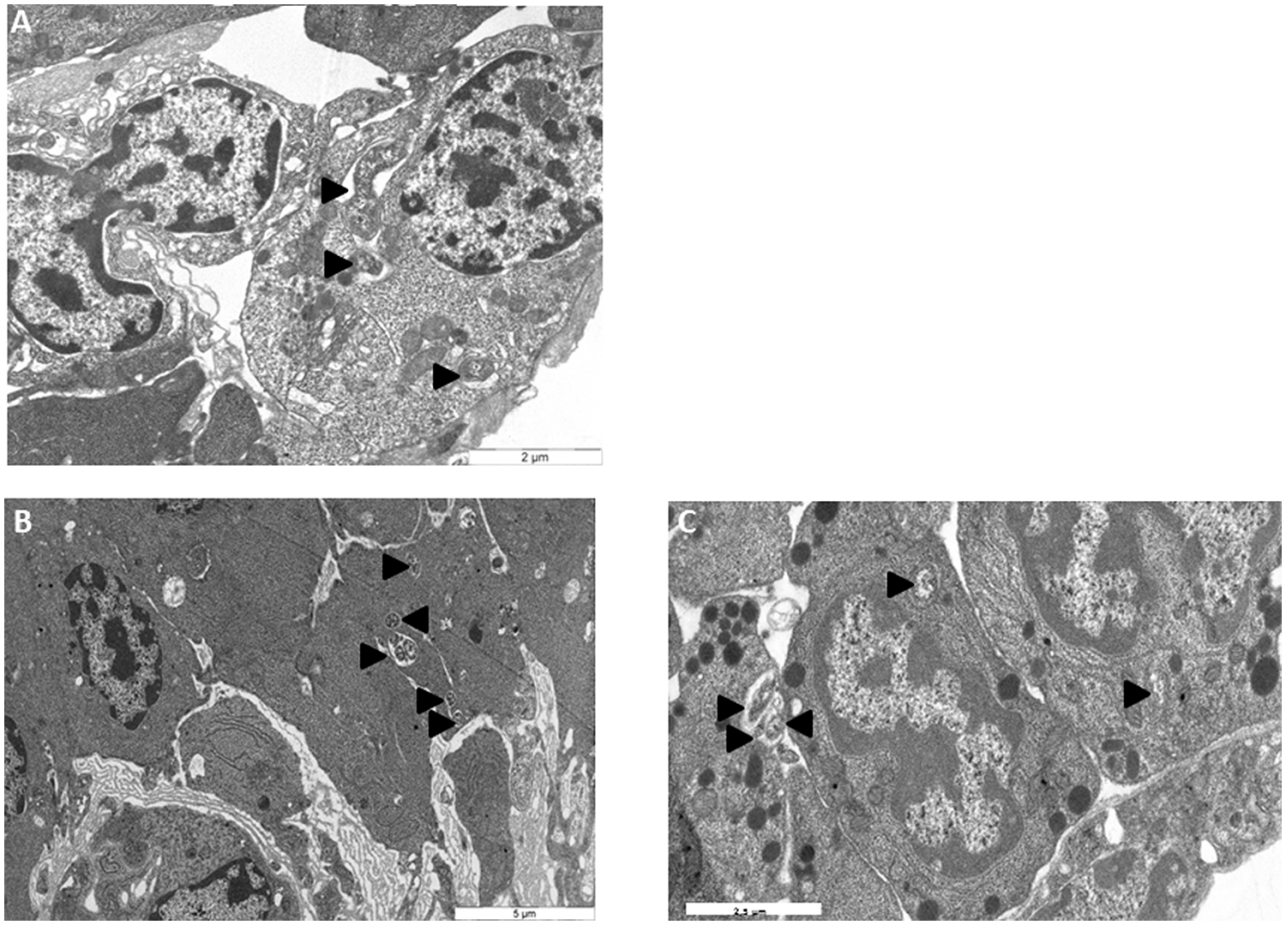
**Transmission electron microscopy observations of asymbiotic HO grafted in symbiotic animals **(A)** 30 days post transplantation **(B,C)** 60 days post transplantation.** H, hemocytes 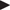 *Wolbachia.*

**FIGURE 5:**
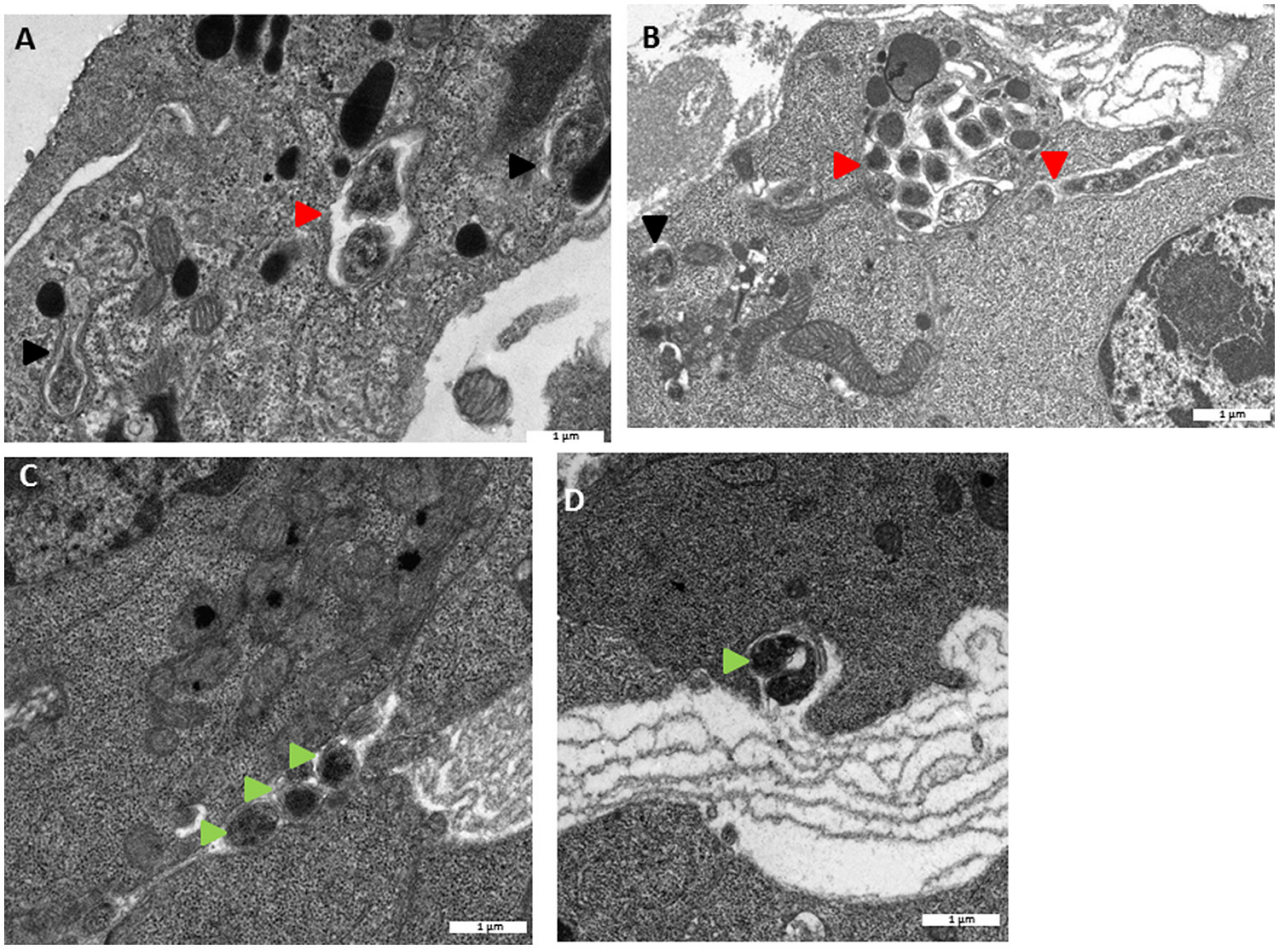
**Transmission electron microscopy observations of *Wolbachia* intracellularly **(A,B)** and extracellularly **(C,D)** in originally asymbiotic HOs 60 days post transplantation.** 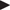 *Wolbachia 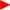 Wolbachia* dividing, 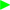 Extracellular *Wolbachia.*

To monitor the behavior of *Wolbachia* in HOs’ cells, HOs coming from 16 naive animals were transplanted to 16 animals infected with *Wolbachia*. The grafted organs were resampled in recipient hosts 15, 30, and 60 days after the transplantation and observed in TEM (**Figures 3**, **4**, and **5**). The transplantation of HOs was used here as an experimental procedure to assess the relevance of this organ as a reservoir for *Wolbachia*. As in all transplantation experiments, there are some limitations in the interpretation as the grafted HOs might have lost their normal function, and could be detected and attacked by the immune response of the recipient. However, we did not observed immune reaction such as encapsulation toward grafted HOs. Starting from 15 days after transplantation, *Wolbachia* were only observed in adipocytes surrounding the HOs but not in the HO itself (**Figure 3A**). Only qPCR allowed the detection of *Wolbachia* at this stage within HOs cells. However, histological observation of HOs showed that the organization of these organs coming from asymbiotic animals changed when they were introduced in symbiotic animals: the compactness of cells is reduced. Such pattern of reduced compactness was also observed in HOs of symbiotic animals. We hypothesize a putative link between colonization by *Wolbachia* and loss of compactness in HOs (**Figures 3B,C**; Chevalier et al., 2011). Then, 30 days after transplantation more cells of the grafted HOs were colonized by *Wolbachia* (**Figure 4A**). The colonized cells were localized at the surface of the HOs. Sixty days after transplantation, all the cells of the transplanted HOs from central area to the surface were infected with several *Wolbachia* cells per host cell (**Figures 4B,C**). We observed *Wolbachia* cells dividing in the same vacuole suggesting a high rate of multiplication in HOs (**Figures 5A,B**). Interestingly, some *Wolbachia* cells appeared to be localized extracellularly between the cells of the HOs suggesting that they could pass from one cell to another in an extracellular manner (**Figure 5C**). These TEM observations showed that naive HOs when transplanted in a symbiotic environment were progressively colonized by *Wolbachia.* The hemocytes produced by the HOs are thus infected with *Wolbachia* (at least 40% of them in Chevalier et al., 2011) and can propagate the infection in the whole organism.

## Discussion

*Wolbachia* is responsible for the most widespread pandemics in the animal kingdom (LePage and Bordenstein, 2013). This secondary facultative symbiont manipulates its hosts reproduction to promote its vertical propagation (Werren et al., 2008). However, for several years, it has been demonstrated that *Wolbachia*, additionally to vertical transmission, can also pass horizontally from one host to another (Sicard et al., 2014). The vision of *Wolbachia* being not only a reproductive parasite has been raised since its impact on many physiological aspects outside reproduction have been revealed (Saridaki and Bourtzis, 2010; Sicard et al., 2014). Previous studies on *Wolbachia*-terrestrial isopod interactions demonstrated that the presence of *Wolbachia* influences the immune phenotype by changing many immune parameters including PO activity, hemocyte density and phagocytosis (Braquart-Varnier et al., 2008; Sicard et al., 2010; Chevalier et al., 2011; Pigeault et al., 2014). Moreover, it has also been demonstrated that the immune cells are infected with *Wolbachia* which (i) live in a vacuole delimitated by several membranes (Braquart-Varnier et al., 2008; Chevalier et al., 2011), (ii) are metabolically active (Chevalier et al., 2011), and (iii) are able to infect new hosts after blood contact (Rigaud and Juchault, 1995; Le Clec’h et al., 2014). For intracellular bacteria, to live inside the cells, is a good way to be protected from both cellular and humoral immune effectors. By living in the immune cells, *Wolbachia*, would not only be preserved from the immune system, but would also benefit from this location to spread. Such ability to colonize actively and survive in immune cells is similar to what was described for *Anaplasma*, a vertebrate pathogen phylogenetically close to *Wolbachia* (Rikihisa, 2010; Pruneau et al., 2014).

**FIGURE 6:**
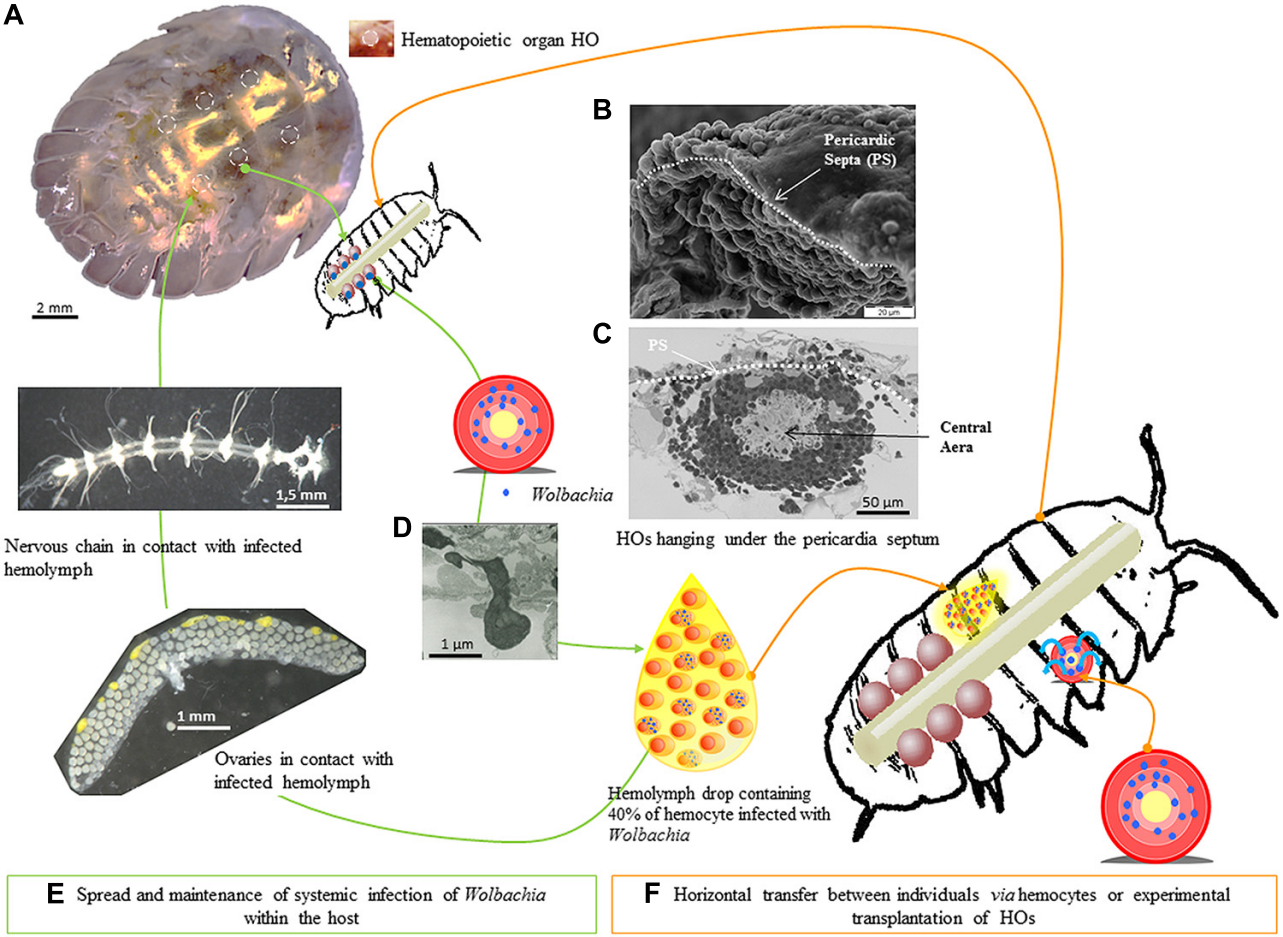
**Hematopoietic organs as cornerstone for *Wolbachia* propagation.** The six hematopoietic organs are localized between the sixth and seventh pereion segments and the first telson segment **(A)**, along the dorsal vessel and hanging under the pericardia septum **(B,C)**. The six HOs were 150 μm long, 150 μm large and 50–60 μm thick **(C)**. Each HO contained only hemocytes presenting different maturation stages, the least mature being in the central area **(C)**. The HOs discharge hemocytes in the hemolymph probably by diapedesis **(D)**. In terrestrial isopod, hemolymph is a vital biological fluid notably implied in the transportation of oxygen and the circulation of immune cells in the general cavity **(E)**. Transmission of *Wolbachia* can also happen when a hemolymph contact occurs between two wounded animals **(F)**.

Our hypothesis is that the localization of *Wolbachia* in hemocytes would be an adaptive feature. Indeed, this colonization can contribute to the maintenance of a systemic infection that could increase vertical transmission of *Wolbachia* by chronic ovary infections (**Figure 6**). In the context of horizontal transmission, the presence of *Wolbachia* in hemocytes constitutes an adaptation for horizontal transfers to new hosts *via* hemolymph contact, a path that should frequently occur *in natura* due to the gregarious behavior of terrestrial isopods and the high frequency of wounded animals (Rigaud and Juchault, 1995; Hornung, 2011). To be able to reach the hemocytes that act as ‘taxis’ for transfer within and among individuals, we suggest that *Wolbachia* could target the hemocyte sources: the HOs. Our empirical data showed that HOs tend to be more colonized than other organs in only a short time after *Wolbachia* introduction.

## Conclusion

As the hemolymph is circulating throughout the isopod body due to the regular contraction of the dorsal vessel, all the isopod organs hosted in the general cavity are permanently in contact with hemocytes (**Figure 6**). As hemocytes are able to traffic between the organ cells such as ovaries, nervous chain, etc., the *Wolbachia* infecting the hemocytes can contaminate cells in many organs including HOs. In this hypothesis, HOs which produce hemocytes all lifelong (up to 3 years for *A. vulgare*) are the cornerstone for the maintenance and propagation of *Wolbachia* between and within terrestrial isopods (**Figure 6**).

## Conflict of Interest Statement

The authors declare that the research was conducted in the absence of any commercial or financial relationships that could be construed as a potential conflict of interest.
